# IS*6110* Restriction Fragment Length Polymorphism Typing of Drug-resistant *Mycobacterium tuberculosis* Strains from Northeast South Africa

**DOI:** 10.3329/jhpn.v31i1.14743

**Published:** 2013-03

**Authors:** Ezekiel Green, Lawrence C. Obi, Anthony I. Okoh, Maphoshane Nchabeleng, Babsie E. De Villiers, Tomas Letsoalo, Anwar A. Hoosen, Pascal O. Bessong, Roland N. Ndip

**Affiliations:** ^1^School of Mathematics and Natural Sciences, Department of Microbiology, University of Venda, Private Bag X5050, Thohoyandou 0960, South Africa;; ^2^Department of Biochemistry and Microbiology, Faculty of Science and Agriculture, University of Fort Hare, Private Bag X1314, Alice 5700, South Africa;; ^3^Division of Academic Affairs, University of Fort Hare, Alice 5700, South Africa;; ^4^Department of Microbiological Pathology, NHLS/University of Limpopo, Medunsa campus, Pretoria, South Africa;; ^5^Department of Medical Microbiology, Faculty of Health Sciences, University of Pretoria/NHLS, Pretoria, South Africa;; ^6^AIDS Virus Research Laboratory, Department of Microbiology, University of Venda, South Africa;; ^7^Department of Microbiology and Parasitology, University of Buea, Box 63, Buea, Cameroon

**Keywords:** Drug resistance, Epidemiology, IS*6110*, *M. tuberculosis*, PCR-RFLP, South Africa

## Abstract

Tuberculosis (TB) remains a deadly infectious disease affecting millions of people worldwide; 95% of TB cases, with 98% of death occur in developing countries. The situation in South Africa merits special attention. A total of 21,913 sputum specimens of suspected TB patients from three provinces of South Africa routinely submitted to the TB laboratory of Dr. George Mukhari (DGM) Hospital were assayed for *Mycobacterium tuberculosis* (MTB) growth and antibiotic susceptibility. The genetic diversity of 338 resistant strains were also studied. DNA isolated from the strains were restricted with *Pvu* II, transferred on to a nylon membrane and hybridized with a PCR-amplified horseradish peroxidase 245 bp IS*6110* probe. Of the 338 resistant strains, 2.09% had less than 5 bands of IS*6110,* and 98% had 5 or more bands. Unique restriction fragment length polymorphism (RFLP) patterns were observed in 84.3% of the strains, showing their epidemiological independence, and 15.7% were grouped into 22 clusters. Thirty-two strains (61.5%) from the 52 that clustered were from Mpumalanga, 16/52 (30.8%) from Gauteng, and 4/52 (9.6%) from Limpopo province. Clustering was not associated with age. However, strains from male patients in Mpumalanga were more likely to be clustered than strains from male patients in Limpopo and/or Gauteng province. The minimum estimate for the proportion of resistant TB that was due to transmission is 9.06% (52-22=30/331). Our results indicate that transmission of drug-resistant strains may contribute substantially to the emergence of drug-resistant tuberculosis in South Africa.

## INTRODUCTION

Tuberculosis (TB) remains a deadly infectious disease affecting millions of people worldwide; 95% of TB cases, with 98% of deaths, occurr in developing countries (1). Approximately one-third of the world's population is infected with tuberculosis, and 2 million people die of the disease every year (2). South Africa is a country with a high incidence of TB—600 cases per 100,000 population in 2005 (3), 550 cases per 100,000 population in 2003, and 718 cases per 100,000 population in 2004 (4,5). During the study period, the cases of drug resistance increased from 156 per 100,000 in 2004 to 177 per 100,000 in 2006 in Mpumalanga, and from 58 to 84 per 100,000 in Limpopo. However, Gauteng showed a high number of drug-resistant cases with an increase from 662 in 2004 to 794 per 100,000 in 2006 (3).

World Health Organization recommends standardized TB treatment regimens based on short-course chemotherapy. The anti-TB drug regimen recommended for the treatment of new cases consists of a two-month administration of isoniazid (INH), rifampicin (RIF), pyrazinamide (PZA), and ethambutol (EMB), followed by a continuation phase of INH/RIF and/or EMB for four months (2). However, this treatment is usually effective against MTB strains that have never been exposed to anti-TB drugs for more than 30 days (6) and against strains that do not possess drug-resistant mechanisms. For many years, Direct Observation of Treatment (DOT) has been promoted by the World Health Organization (WHO) as one of the five components of a wider strategy called Directly Observed Treatment, Short Course (DOTS) to tackle the resurgence of TB throughout the world (7). Direct observation by health workers while patients take their tablets aims to improve adherence to therapy and completion of treatment. Specific therapy for patients with drug-resistant tuberculosis is included in DOTS-plus. A surge in drug-resistant tuberculosis in several parts of the world requires effective implementation of the DOTS-plus strategy to prevent the occurrence of new multidrug-resistant (MDR) TB cases and to reduce transmission of MTB.

Resistance to antibiotics in MTB occurs due to genomic mutations in certain genes, such as *kat*G for INH resistance and *rpo*B for RIF resistance (8). Therefore, MTB will benefit from increased mutation rate. Unlike other pathogens with MDR pathotypes, such as transposable elements, plasmid-mediated mechanisms of resistance have not been reported in MTB (9,10).

In recent years, treatment of TB has become complicated by increasing emergence of drug-resistant *M. tuberculosis* (DR-TB) (11). The proportion of MDR-TB strains in South Africa rose from 1.1% in 2004 to 1.9% in 2006 (3). Extremely drug-resistant (XDR) TB strains have also been reported in the country (12).

Analysis of the spread and transmission of DR-TB strains, using molecular methods, has been reported (13). Techniques, such as restriction fragment length polymorphism (RFLP) and insertion sequence 6110 (IS*6110*), have been used in reliably differentiating *M. tuberculosis* isolates (14,15), and IS*6110* has been suggested as a standard tool for characterization of isolates. IS*6110* fingerprinting has also been used successfully to confirm laboratory cross-contaminations and to trace small-scale outbreak of TB and DR-TB in a large variety of settings (16). Although research on MTB transmission, using molecular techniques, has been conducted in the Western Cape (17), Kwazulu-Natal (18,19), Gauteng, and North West (20) province, similar information has not been recorded for DR-TB isolates from Gauteng, Limpopo, and Mpumalanga provinces. There is, therefore, a paucity of information on epidemiological data based on molecular methods addressing the transmission routes of DR-TB strains. This merits attention considering that the country has a high prevalence of HIV/AIDS, a confounding factor for TB resurgence.

The aim of this study was to determine the genetic diversity of resistant MTB isolates, using IS*6110* to delineate the dissemination of major phylogenetic clades of the organism in Gauteng, Limpopo, and Mpumalanga regions of South Africa.

## MATERIALS AND METHODS

### Study location and population

The present study was conducted in the TB Laboratory of the DGM Hospital, Gauteng province of South Africa. This province showed a TB prevalence rate of 500/100,000 population in 2006 (21). From January 2004 to December 2006, sputum specimens of all patients ((n=21,913) routinely sent for TB analysis to microbiology laboratory of DGM Hospital were included in the study. During the study period, we were aware of only three provinces that had TB diagnostic laboratories, which included Gauteng, Western Cape, and Kwazulu-Natal. We concentrated on DGM Hospital since it received specimens from other provinces, including Limpopo and Mpumalanga, without diagnostic centres.

### Patients' characteristics

The samples used were routinely collected from patients by hospitals and sent to DGM Hospital for TB diagnosis. Data on the patients, concerning type of specimens and standard demographic information (gender, age, and province), were registered. The study was approved by the Research and Ethics Committee of the University of Venda, South Africa. Informed consent was not obtained from the subjects.

### Bacterial isolates

All specimens were processed and cultured in the BacT/Alert 3D (BioMérieux, Durham, NC, USA) system. Positive cultures were stained with Ziehl Neelsen and confirmed with the AccuProbe DNA hybridization assay (Gen-Probe, USA) according to the manufacturer's instructions. Isolates were then advanced for susceptibility testing and sequencing of *kat*G and *rpo*B genes (22,23). The isolates of MTB analyzed in this study represent all available isolates obtained from patients of Limpopo, Mpumalanga, and Gauteng province, attending DGM Hospital between January 2004 and December 2006. The TB laboratory of DGM Hospital serves as one of the major TB laboratories situated in Gauteng province of South Africa. All the isolates were examined for their susceptibility to isoniazid (INH), ethambutol (EMB), streptomycin (SM), and rifampicin (RIF) following instructions of MGIT 960 system (MGITs; Becton Dickinson Microbiology systems, Sparks, MD, USA). Three hundred thirty-eight resistant strains of *M. tuberculosis* were obtained from 21,913 patients between January 2004 and December 2006. Of all the resistant strains obtained, 97 (28.7%) were from Gauteng, 32 (9.5%) from Limpopo, and 209 (61.8%) from Mpumalanga province. The provinces share borders with one another, with Mpumalanga situated in the Southeast of Limpopo and East of Gauteng, and Limpopo in the North of Gauteng.

### Extraction of DNA

The MGIT 960 cultures were heat-inactivated at 80 °C for 1 hour before DNA extraction was performed in a biosafety level 2 laminar flow cabinet as previously described (22,24). Briefly, growth from the MGIT 960 system was suspended in 6 mL of DNA extraction buffer (5% monosodium glutamate, 50 mM Tris-HCl, pH 7.0 and 25 mM EDTA) in a sterile 50-mL polypropylene tube which contained approximately thirty 5-mm glass balls. The bacterial clumps were disrupted by vigorous shaking and vortexing. Five hundred microlitre of lysozyme (Amersham Biosciences, Greece) and 10 μL of RNAseA (Amersham Biosciences, Greece) were added to the tube. The contents of the tube were mixed by gentle inversion and then incubated at 37 °C for 2 hours. After incubation, 600 μL of 10×Proteinase K buffer and 150 μL of Proteinase K (Amersham Biosciences, Greece) were added. The sample was gently mixed (inverting the tube a few times) and then incubated overnight at 45 °C. Proteins were removed by phenol/chloroform and chloroform/isoamyl-alcohol extraction method (25). DNA was then precipitated with the addition of 600 μL of 3 M sodium acetate (pH 5.5) and 7 mL of cold (-20 °C) isopropanol. The precipitated DNA was washed with 1 mL of 70% ethanol for approximately 1 minute. DNA was then air-dried and resuspended in 30 µL in TE buffer (10 mM Tris, 1 mM EDTA, pH 8.0).

### Typing of *M. tuberculosis* strains, using IS*6110*

DNA was extracted using the phenol/chloroform method as described earlier (24,25). RFLP was performed using the standardized IS*6110* technique as described previously (15). The extracted genomic DNA was restricted with *Pvu* II (20) in a reaction mix (final volume 30 mL) consisting of 3 μg of genomic DNA, and 15 units of *Pvu* II in 3 μL of the prescribed restriction buffer (Amersham biosciences, Greece). The restriction mix was incubated overnight (±16 h) at 37 °C. At the end of digestion, the reaction was incubated at 65 °C for 10 minutes to inactivate any remaining enzyme activity. Restricted products were resolved on a 0.8% agarose at constant voltage of 40 in 1X TBE buffer for 24 hours. The probe used for hybridization was generated by PCR, using primers INS1 (5 CGT GAG GGC ATC GAG GTG GC 3) and INS2 (5/ GCG TAG GCG TCG GTG ACA AA 3/) labelled with horseradish peroxidase (ECL^tm^ direct nucleic acid labelling and detection kit, Amersham, UK); hybridization and detection were carried out as per manufacturer's instructions.

We restricted the analysis to the isolates with 5 or more bands because isolates with few or no copies of the IS*6110* element cannot be reliably classified by this method (26). IS*6110* fingerprints were analyzed visually as described earlier (27). Recent transmission was considered likely if an isolate matched at least one other by identical or near-identical criteria. ‘Identical’ isolates were characterized by equal number of bands on gel electrophoresis, following digestion by restriction endonuclease; all such bands had to have matching molecular weights. ‘Near-identical’ isolates were characterized by difference of a single band (addition or loss of a single band). RFLP patterns were grouped from 1 to 20 based on the number of IS*6110* band. A cluster was defined as a group of two or more patients with drug-resistant MTB strains whose fingerprints were identical with respect to both number and the size of all bands. We used the n-1 method to estimate continuing transmission. The method is based on the assumption that one case per cluster is due to reactivation and that this ‘index’ infectious case gives rise to other cases in the cluster either by infecting them directly or infecting a secondary case that then infects other members of the cluster. It calculates the number of recently-transmitted cases by summing within clusters after reducing each cluster-size by one giving the transmission dynamics (28). It is calculated by the following formula:

Total number of strains in clusters [Number of clusters]/Total number of strains

## RESULTS

### Study location and patients' characteristics

DGM Hospital is a 1,200-bed tertiary referral hospital attached to the Medunsa Campus of the University of Limpopo. HIV status was confirmed for only 64 patients from the hospital-records.

### Bacterial isolates

A total of 1,648/21,913 (7.5%) MTB isolates were obtained from sputum specimens cultured during the study period. Furthermore, 338/1,648 (20.5%) resistant strains of MTB from the 1,648 culture-positive strains were obtained. Of these, 97/338 (28.7%) were from Gauteng, 32/338 (9.5%) from Limpopo, and 209/338 (61.8%) from Mpumalanga province. Of the isolates, 33 (63.5%) were MDR, 6 (11.5%) resistant to two drugs, 2 (3.8%) resistant to three drugs, and 11 (21.1%) resistant to one drug.

### IS*6110* RFLP clustering

**Figure 1.**
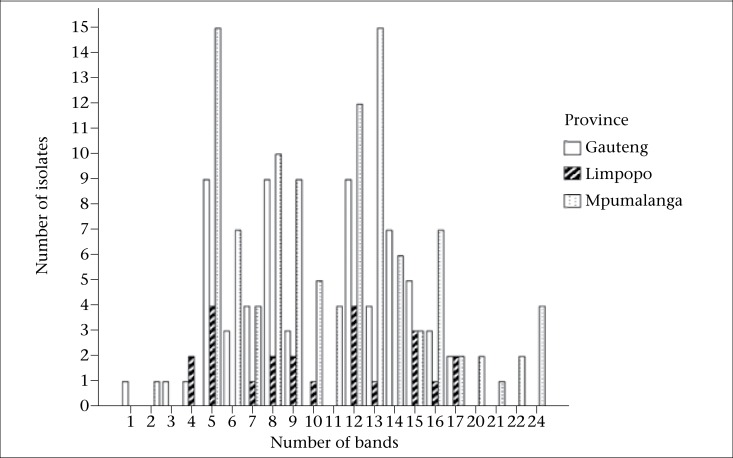
Frequency of MTB isolates from male patients with different copy numbers of the IS*6110* elements

**Figure 2.**
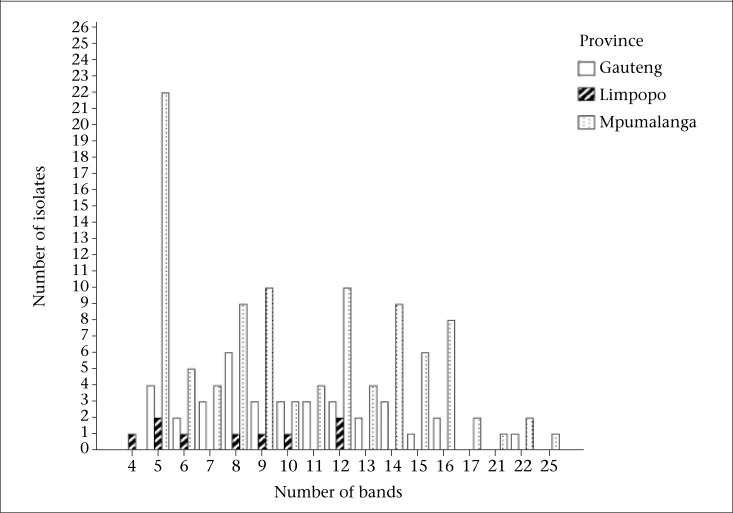
Frequency of MTB isolates from female patients with different copy numbers of the IS*6110* elements

The copy number of IS*6110* in each of the isolates was determined from the number of bands hybridizing with the probe, and the copies varied from 1 to 24. Seven of the 338 strains (2.07%) had less than 5 bands. Most strains (331/338, 97.9%) carried 5 or more copies of IS*6110* (Figure 1 and 2). Of the 331 strains, 279 (84.3%) had unique RFLP patterns. Twenty-two clusters (designated A-V) made up of 52 (15.7%) strains were observed (Table). Thirty-two stains (61.5%) of the clustered 52 were from Mpumalanga, 16/52 (30.8%) from Gauteng, and 4/52 (7.7%) from Limpopo province. The minimum estimate for the proportion of resistant TB that was due to transmission is 9.06% (52-22=30/331)x100 while the diversity was 90.9% (279+22/331)x100 as previously defined (29).

### Epidemiological investigation of fingerprint groups

The largest cluster comprised seven patients with drug-resistant TB strains, of which 4 were from Mpumalanga, 2 from Gauteng, and 1 from Limpopo. Five of these patients were male, and two were female. The second cluster was made up of 4 DR-TB strains: two each were isolated from patients who were from Mpumalanga and Limpopo province. One cluster harboured three DR-TB strains isolated from male patients, of whom two were from Mpumalanga and one from Gauteng. The remaining 19 clusters comprised two DR-TB strains isolated from patients who were from Gauteng, Mpumalanga and Limpopo province. The ages of the patients ranged from 10 to 69 years (Table).

### Relationship of cluster and resistance

**Figure 3.**
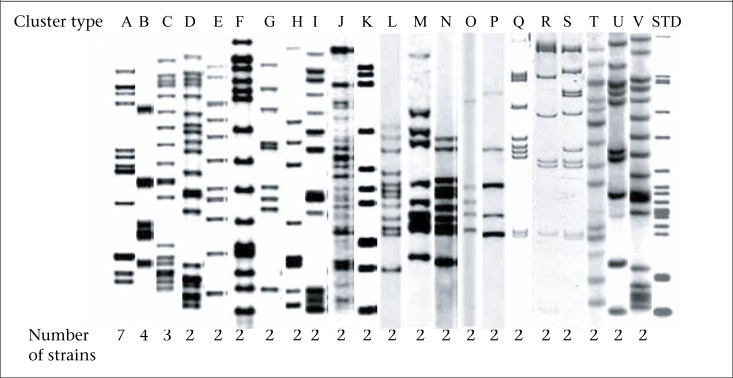
Representative examples of IS*6110* RFLP patterns associated with 22 high-copy clusters (A-V). The number of strains in each cluster is shown at the bottom of each pattern. The last lane shows reference strain

The drug-resistance patterns of the strains clustered in different fingerprint groups were not similar. The highest number of bands corresponding to 20 was noted for cluster D while the lowest made up of 5 bands occurred in strains from cluster O and P. However, band-sizes of 7, 9, 10, 11, 12, 14, 15, 18, and 19 were also noted. There were differences in the sizes of the individual bands (Figure 3).

### PCR and sequencing

Sequencing of the PCR products on the *rpo*B gene hotspot region showed nucleotide substitution at codon 516 (GAC to GTC) in 44.7% (72/161) and codon 526 causing a codon change at CAC to GTC in 50.3% (81/161) of 47.6% (161/338) RIF-resistant isolates studied. One isolate (0.6%, 1/161) showed the mutation in codon 512 AGC to ACC, 515 ATG to CAT, 516 GAC to GTC, 517 CAG to CCA, 518 AAC to GAA, 519 AAC to CAA, 525 ACC to CAC, 526 CAC to GAC, 529 CGA to CCC, 530 CTG to CCG, 531 TCG to TTG, 532 GCG to CGG, and 533 CTG to CGC in the *rpo*B region. Overall, 17 different mutations were observed: 29.4% (5/17) single, 64.7% (11/17) double, and 5.9% (1/17) triple mutations.

In total, 60.3% (204/338) MTB isolates were resistant to INH. Sequencing of the INH-resistant isolates showed a nucleotide substitution at codon 315 in 90.1% (184/204) of the resistant isolates, of which 125/184 (67.9%) had a codon change from AGC to ACC, 38/184 (20.6%) had a change from AGC to AAC, and 21/184 (11.4%) changed from AGC to ACA. A mutation at codon 314 (ACC to CCC) of 20/204 isolates contributed 9.8% of INH resistance (Table).

## DISCUSSION

IS*6110*-based DNA fingerprinting of MTB has proven to be highly effective in detecting the source of infection and route of transmission and in simplifying diagnosis of the outbreak (30). In this study, we utilized IS*6110* to determine the genetic diversity of drug-resistant MTB isolates from the DGM Hospital in South Africa.

Of the 338 resistant strains (209 from Mpumalanga, 32 from Limpopo, and 97 from Gauteng), 7 (2.07%) showed fewer than 5 copies of IS*6110*. Similar results (2.39%) were obtained in isolates from Germany (31), 5% in isolates from Rio de Janeiro, Brazil (15), and 8% strains from East Azerbaijan, Iran (32). However, the number of strains with low-copy number of IS*6110* in our study is lower (2.07%) than that observed in regions, such as Poland-12% (33), Delhi, India-18.3% (34), and Germany-11.9% (29). Many reports on epidemiological studies of TB use a secondary typing to increase the accuracy of indicating epidemiological links. In the case of IS*6110*-RFLP patterns, this is considered necessary, mainly when dealing with MTB populations presenting a high proportion of strains with low-copy number (35). In light of these findings, IS*6110* can be used without additional typing markers for this area.

**T1able:** Fifty-two drug-resistant *M. tuberculosis* grouped into 22 IS*6110* clusters

Group	Gender	Age	Drug resistance pattern	*kat*G	*rpo*B	Province
A	Male	33	INH, RIF	Thr315	Thr512, Val516	Mpumalanga
	Male	25	INH	Thr315	WT	Mpumalanga
	Female	35	INH, RIF, EMB, SM	Arg315	Gly516, Asp526	Mpumalanga
	Female	26	INH, RIF, EMB, SM	Arg315	Gly516, Asp526, Tyr522	Mpumalanga
	Male	47	EMB	WT	WT	Gauteng
	Male	29	INH, RIF, EMB, SM	Asn315	His525, Tyr522	Gauteng
	Male	30	INH, RIF, EMB	Thr315	His525	Limpopo
B	Male	53	SM	WT	WT	Mpumalanga
	Male	26	INH, RIF, EMB, SM	Thr315	Thr512, Asp526, Tyr522	Mpumalanga
	Male	51	INH	Ile315	WT	Limpopo
	Female	34	INH, RIF	Ile315	Thr512, Asp526, Arg532	Limpopo
C	Male	18	INH, RIF, EMB, SM	Thr315	Gln518	Mpumalanga
	Male	60	INH, RIF, EMB	Arg315	Asp526	Mpumalanga
	Male	29	INH, RIF, SM	Thr315	Thr512, His515, Val516	Gauteng
D	Male	37	INH, RIF, SM	Arg315	Thr512, His515, Val516	Mpumalanga
	Male	54	INH, RIF, EMB, SM	Pro314,Thr315	Gly516	Mpumalanga
E	Female	18	INH, RIF, SM	Thr315	Thr512, Val516	Gauteng
	Female	69	SM	WT	WT	Limpopo
F	Male	41	INH, RIF, EMB, SM	Arg315	Thr512, Asp526, Arg532	Limpopo
	Male	44	INH	Arg315	WT	Mpumalanga
G	Male	35	SM	WT	WT	Mpumalanga
	Female	37	INH, SM	Thr315	WT	Gauteng
H	Male	44	INH, SM	Thr315	WT	Gauteng
	Male	33	INH, RIF, EMB, SM	Pro314	Gly516, Arg532	Mpumalanga
I	Female	22	INH, RIF, EMB	Thr315	Arg533	Gauteng
	Male	29	INH	Thr315	WT	Mpumalanga
J	Male	33	INH, RIF, EMB, SM	Thr315	Thr512, Asp526, Arg532,	Mpumalanga
	Male	42	INH, RIF	Pro314	Gly516, Arg533	Mpumalanga
K	Female	32	INH	Pro314	WT	Mpumalanga
	Male	10	INH, RIF	Thr315	Pro529	Gauteng
L	Male	50	INH	Thr315	WT	Mpumalanga
	Male	31	INH, RIF	Pro314	Thr512, Arg532, Gln518	Mpumalanga
M	Male	21	INH, RIF, EMB, SM	Asn315	Glu519	Mpumalanga
	Male	41	INH, EMB, SM	Thr315	Pro529, Glu519	Mpumalanga
N	Male	34	EMB, SM	WT	WT	Gauteng
	Male	26	INH, RIF, EMB, SM	Arg315	Gln518, Cys531	Mpumalanga
O	Male	33	INH, RIF, EMB, SM	Thr315	Pro530, Tyr522	Mpumalanga
	Female	50	INH, RIF	Thr315	Thr512, Pro529, Glu519	Mpumalanga
P	Male	54	INH, RIF, SM	Pro314	Pro529, Glu519	Mpumalanga
	Male	30	INH, EMB, SM	Thr315	Gln518, Cys531	Mpumalanga
Q	Male	33	INH, RIF, EMB,	Thr315	Tyr522	Mpumalanga
	Male	28	INH, RIF, EMB, SM	Thr315	Gly516, Cys531	Mpumalanga
R	Female	18	INH, RIF	Thr315	Asp530	Gauteng
	Male	60	INH, RIF, EMB	Thr315	Arg532	Mpumalanga
S	Male	31	INH, SM	Asn315	His515	Mpumalanga
	Female	45	INH	Thr315	Val516, Gln518, Glu519	Gauteng
T	Male	37	INH, RIF, SM	Thr315	Asp526, Arg532, Arg533	Mpumalanga
	Male	31	INH, RIF	Asp315	Val516, Asp526	Mpumalanga
U	Male	29	INH, RIF, EMB, SM	Pro314	Asp526, Gln518	Gauteng
	Male	37	INH, SM	Thr315	Pro529	Gauteng
V	Female	21	INH, RIF, EMB, SM	Thr315	Arg532	Mpumalanga
	Female	34	INH, EMB	Thr315	WT	Mpumalanga

Recent population-based studies have shown that patients with MTB strains showing identical IS*6110* fingerprint patterns are likely to have become infected recently (36). In our study, 22 fingerprint groups with identical patterns were found. Most of these patterns consisted of 2 patients, indicating that tuberculosis transmission in this region may be described as a micro-epidemic. In the past two decades, the rare occurrence of drug-resistant strains was ascribed to the conviction that drug-resistant MTB strains were less virulent (37). However, studies have shown that drug-resistant strains do not differ from drug-sensitive strains in their ability to create infection or disease, and that drug-resistant strains contribute substantially to the increase of tuberculosis (38-41).

Mpumalanga is one of the poorest provinces in South Africa, and people living in the province are of low socioeconomic status (42). MDR-TB cases of 1.99% were reported in the province in 2004 (3). The cases increased to 2.64% in 2006 and decreased drastically to 1.17% in April 2007. This is highly worrisome as it may mean that many patients with MDR-TB are not being diagnosed. This region might be a risk-zone because most (65.4%) of the clustered isolates were obtained from this zone compared to the other investigated provinces. This corroborates previous works in which the region was defined as a risk-zone for tuberculosis clustering (43). However, clustering could have been overestimated as more samples analyzed (61.8%) were from Mpumalanga than from Gauteng and Limpopo while other provinces were not investigated. Gauteng is the richest province in South Africa (44). This province showed an incidence rate of 500/100,000 in 2006 compared to 722/100,000 for overall South Africa.

In a study done in the same laboratory, Rampe *et al.* (20) reported a total number of clustering of 0.47, indicating a transmission rate of 47%. However, that study focused on isolates from the locations surrounding DGM Hospital (previously called Ga-Rankuwa Hospital), and the study had a lower sample-size. We observed a 52-22=30/331 (0.096) clustering among resistant *M. tuberculosis* isolates, indicating recent transmission rate of about 9%. We also observed that, besides failures of tuberculosis treatment, transmission of drug-resistant tuberculosis contributes to the problem of drug resistance in South Africa. At least 91% of the problem of drug resistance in this population could be the product of acquired resistance. However, in the absence of clinical data (retreatment or primary resistance) on the patients from whom these strains were isolated, it is difficult to conclude whether they represent fresh cases or cases of reactivation. In a mixed population, the degree of DNA polymorphism is greater (15)—a situation common in South Africa. In South Africa, people also travel to other provinces looking for jobs, which may introduce new strains into otherwise unaffected areas, leading to high genetic diversity.

In our study, strains that clustered demonstrated resistance to the same antitubercular drug with or without resistance to additional drugs. The likelihood is that an additional resistance was acquired after transmission occurred. The close relationship of the strains clustered in different groups (e.g. A, O, and P) was further confirmed by the overall good association between IS*6110* and data on drug resistance. This report of clustered strains further confirms that drug-resistant MTB isolates can be transmitted. Furthermore, it should be noted that the study was performed using isolates obtained from one hospital and because of disease transmission which emanated from the clusters and the time it takes for MTB to grow (6-8 weeks); a study using a larger population should be performed to evaluate the real clustering rate.

One of the risk factors identified in our study was the male gender. Of the 52 clustered strains, 39 (75%) were from the male gender. A study in Paris showed the same results (45). However, a study in Iran showed more clustering in females than in males (30). Although few epidemiological links were confirmed in this study, demographic and behavioural risk factors, such as body mass index, alcohol consumption, and smoking, have been identified as the causes of tuberculosis clustering elsewhere (46). This is understandable as smoking and alcohol consumption may be proxies for frequenting locations that puts one at risk of close contact with infectious individuals, such as neighborhood bar (46).

Being a pro-drug that requires activation by catalase peroxidase enzyme produced by MTB, INH plays an important role in treating latent MTB infection (LTBI), for prevention of active disease and the subsequent TB transmission. It is also a cornerstone of the modern short-course chemotherapy for tuberculosis. Mutation in the gene producing this enzyme will render the organism resistant to INH. Codon 315 mutations were shown to be associated with high-level resistance to INH. Results obtained from this study suggest that serine substitution at codon 315 of the *kat*G gene (57.7%) is a characteristic of local INH-resistant strains and, therefore, can serve as a genetic marker for INH-resistance in our region. The high proportion of *kat*G 315Thr-resistant isolates in this study had mutation in the *rpo*B gene (46%), which was related to RIF resistance reported in South Africa (21).

### Conclusions

Our results indicate that transmission of drug-resistant strains may contribute to the emergence of drug-resistant tuberculosis in South Africa—a finding with profound clinical and epidemiological significance.

## ACKNOWLEDGEMENTS

We would like to thank the National Research Foundation of South Africa for providing financial support for the study, the staff members at DGM Hospital for making available their facilities and technical assistance.
